# On the Fitness Functions Involved in Genetic Algorithms and the Cryptanalysis of Block Ciphers

**DOI:** 10.3390/e25020261

**Published:** 2023-01-31

**Authors:** Osmani Tito-Corrioso, Mijail Borges-Quintana, Miguel A. Borges-Trenard, Omar Rojas, Guillermo Sosa-Gómez

**Affiliations:** 1 Departamento de Matemática-Física Aplicada, Facultad de Ingeniería Industrial, Universidad de Matanzas, Autopista a Varadero km 3.5, Matanzas 40100, Cuba; 2 Departamento de Matemática, Facultad de Ciencias Naturales y Exactas, Universidad de Oriente, Av. Patricio Lumumba s/n, Santiago de Cuba 90500, Cuba; 3 Doctorate in Mathematics Education, Universidad Antonio Nariño, Bogotá 111321, Colombia; 4 Facultad de Ciencias Económicas y Empresariales, Universidad Panamericana, Álvaro del Portillo 49, Zapopan 45010, Mexico; 5 Faculty of Economics and Business, Universitas Airlangga, Jl. Airlangga No. 4–6, Surabaya 60286, Indonesia

**Keywords:** genetic algorithm, fitness function, block ciphers, cryptography, optimization

## Abstract

There are many algorithms used with different purposes in the area of cryptography. Amongst these, Genetic Algorithms have been used, particularly in the cryptanalysis of block ciphers. Interest in the use of and research on such algorithms has increased lately, with a special focus on the analysis and improvement of the properties and characteristics of these algorithms. In this way, the present work focuses on studying the fitness functions involved in Genetic Algorithms. First, a methodology was proposed to verify that the closeness to 1 of some fitness functions’ values that use decimal distance implies decimal closeness to the key. On the other hand, the foundation of a theory is developed in order to characterize such fitness functions and determine, a priori, if one method is more effective than another in the attack to block ciphers using Genetic Algorithms.

## 1. Introduction

There is a plethora of algorithms used in cryptography, with different purposes; amongst them, Genetic Algorithms (GAs) have received an increased focus of attention, as can be observed from the number of recent publications on the subject. GAs have been applied to different areas of science. For example, in [1], the authors discussed various methods to find approximate solutions to the TSP problem (Traveling Salesman Problem), and they proposed a modification of GAs to solve the problem of streamlining the shipping route. In [2], a method based on GAs for processing and classifying electroencephalogram signals was proposed. In [3], a combination of GAs with neural networks was applied to electronic commerce. Other applications can be found in [4,5,6,7], amongst many others.

In recent years, the use of GAs in cryptography has increased, particularly within cryptanalysis, intending to find an optimal solution (the so-called key) within the key space and one that is as close as possible to the real key. Some of the works in this direction are the following: In [8], the authors applied GAs to the cryptanalysis of the RSA (*Rivest, Shamir, and Adleman*) cipher. Something similar was done in [9], where GAs were used to look up factors of the RSA public key. According to the authors, the research results suggest that GAs can break the RSA encryption’s public key. In [10], the authors proposed an attack method inspired by GAs based on the collateral channel attack. One of the algorithms to which they applied this tool was DES (*Data Encryption Standard*).

In [11], a hybrid tool was developed that creates ciphertexts from the combination of GAs and the Particle Swarm Optimization algorithm. Shannon’s Entropy method was used as a fitness function in both algorithms. The authors claimed that the proposed application offers an alternative data encryption and decryption method that can be used to transmit messages. In [12], a technique for encrypting texts based on the mutation and crossing operations of GAs was presented. The proposed encryption technique consisted of dividing the plaintext characters into parts and applying the crossover operation between them, followed by the mutation operation to obtain the ciphertext. In [13], the authors discussed comparing traditional cryptographic algorithms and GA-based cryptosystems.

For more details on the structure and values of the parameters and operators of GAs, which are used in the experiments presented in this article, see [14]. More details on the use of GAs in cryptography can be seen, for example, in [15,16,17,18,19].

Other investigations are directed to the analysis and improvement of the properties and characteristics of the GAs. An example of the above is [14], where several aptitude functions are proposed, and through some experiments, it was studied which of these functions provide the best results in the application of GAs; thus, it has been possible to appreciate the scarcity of theoretical results that can be used in such analysis. On the other hand, there is also the problem of analyzing whether the closeness to 1 of the fitness functions that use decimal distance implies decimal closeness between the new element found and the real key. In this sense, in the present work, a study was conducted on the fitness functions that intervene in GAs with the aim of improving their properties. So our contributions are: (1) a methodology to verify that the closeness to 1 of the values of some fitness functions that use decimal distance implies decimal closeness to the key; (2) a block cipher attack methodology based on the results of (1); and (3) the foundation of a theory that allows us to characterize fitness functions and determine, a priori and from a theoretical point of view, if one fitness function is more efficient than another in attacking block ciphers.

## 2. Preliminaries

### 2.1. Genetic Algorithm

We assume that the reader is familiar with the general ideas of how some heuristic optimization methods work. This section briefly describes the GAs scheme used in this work.

In Algorithm 1, the population’s individuals will be elements of the key space taken as binary blocks. By Selecting the *s* parents, a subset *S* of $P_{i}$ is obtained. These parents are selected by the Tournament Method between two, selecting two individuals randomly and choosing the one with the highest aptitude. Elements of *S* are crossed, and descendants are added to $P_{i}$ if they are not members. For Crossover, the two-point crossover will be used, and the probability of two individuals crossing-over was set to 0.6 for all experiments. The Mutate operation changes at most three binary block’s random components, with a mutation ratio set to 0.2 in all experiments. An individual *x* is better adapted than another individual *y* if it has greater fitness, i.e., if F(x)>F(y).

The application of GAs for cryptanalysis presented in this work uses a known plaintext–ciphertext attack, in which the attacker knows a set of plaintexts with their corresponding encrypted texts. The attack aims to find the key with which the plaintexts were encrypted.
**Algoritmo 1** Genetic Algorithm.**Input:***m* (number of individuals in the population), *F* (fitness function), *g* (number of generations), *s* (number of individuals selected to mate). **Output:** the individuals with the highest fitness function as best solution.1:**Generate** randomly an initial population $P_{i}$ with *m* individuals.2:**Calculate**F(x), $\forall x\in P_{i}$ (the fitness of each individual of $P_{i}$).3:**while** no solution found or *g* generations not reached **do**4:   **Select** *s* parents of $P_{i}$.5:   Apply the **Crossover** operator to the *s* selected elements and generate offspring pairs.6:   **Mutate** each of the resulting descendants.7:   **Compute** the fitness of each of the offspring and their mutations with *F*.8:   Using the Tournament Method between two, based on the aptitudes of the parents and offspring, decide what will be the new population $P_{i+1}$ for the next generation, selecting two individuals at random each time and choosing the higher fitness.9:**end while**


### 2.2. Fitness Functions

The focus of this paper will be fitness functions. In particular, the following functions will be used. Let

(1) $$E:F_{2}^{{}^{m}}\times F_{2}^{{}^{n}}\to F_{2}^{{}^{n}},$$

with $m,n\in Z_{+}^{{}^{*}}$ and m≥n, be a block cipher, *T* a plaintext, *K* a key and *C* the corresponding ciphertext, i.e., C=E(K,T). The first fitness function based on Hamming’s distance between binary blocks, $d_{H}$, for a certain individual *X* of the population, is:

(2) $$F_{1}\left(X\right)=\frac{n-d_{H}\left(C,E\left(X,T\right)\right)}{n},$$

which measures the distance between the ciphertext *C* and the text obtained from encrypting *T* with the probable key *X*.

The following fitness function is based on measuring the distance between plaintexts but on their representation in decimal and not binary. Let $Y_{d}$ be the corresponding conversion to decimal of the binary block *Y*. Then, we have:

(3) $$F_{4}\left(X\right)=\frac{2^{n}-1-\left|C_{d}-E{\left(X,T\right)}_{d}\right|}{2^{n}-1}.$$

Note that if the ciphertexts are equal, i.e., $C_{d}=E{\left(X,T\right)}_{d}$, then $F_{4}\left(X\right)=1$. I.e., if they are equal, then the fitness function takes the highest possible value. On the contrary, the greatest difference is the farthest they can be, e.g., if $C_{d}=2^{n}-1$, and $E{\left(X,T\right)}_{d}=0$, then $F_{4}\left(X\right)=0$. For more details on these fitness functions and other proposals with similar ideas, see [14], where $F_{1}\left(X\right)$ and $F_{4}\left(X\right)$ appear with the same name. Regarding fitness functions and GAs, take into account that an individual *x* of the population is better adapted than another, *y*, if it has greater fitness, i.e., if F(x)>F(y).

### 2.3. Partitioning the Key Space

In this article, two key space partitioning methodologies are used, BBM and TBB (the names of the methodologies come from the authors’ last names’ initials, see the appendix), which allow GAs to work on a certain set of keys’ subset, with admissible solutions as if it was the complete set. This form of partitioning into equivalence classes allows for GAs to be used in parallel, independent and simultaneously, in several classes.

In what follows, a brief description of both methodologies is given; for more details see [14,20]. Let $F_{2}^{k_{1}}$ be the space of keys of length $k_{1}\in Z$, $k_{2},k_{d}\in Z_{>0}$, such that, $1\leq k_{2}<k_{1}$, $k_{d}=k_{1}-k_{2}$, and, $Q=\{0,1,2,\ldots ,2^{k_{d}}-1\}$. So, in both methodologies, the formulas to represent the elements of $F_{2}^{k_{1}}$ are identical:

(4) $$q\;2^{k_{2}}+r,\;q\in Q,\;r\in Z_{>0}.$$

This equation can be used to summarize the differences between these methodologies. Both consist of keeping the GAs running on a subset of the key space rather than the entire key space. In the case of BBM, the subset is associated with the class of keys that correspond to the same quotient (*q*). The TBB methodology consists of working with the subset given by the keys with the same remainder (*r*); the elements of each class are scattered throughout the set of keys.

**Figure 1:**
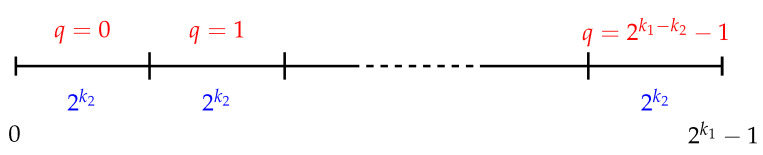
Graphic scheme of the BBM methodology.

In the case of the BBM methodology, the idea of the division made in the keys’ space can be seen in the diagram in Figure 1, where the one-to-one correspondence is assumed between $F_{2}^{k_{1}}$ and the interval $\left[0,2^{k_{1}}-1\right]\subset Z_{+}$. Note that *q* determines the interval and *r* the position of the element in that interval, then all $n\in [0,2^{k_{1}}-1]$ are represented as $n=q2^{k_{2}}+r$.

**Figure 2:**
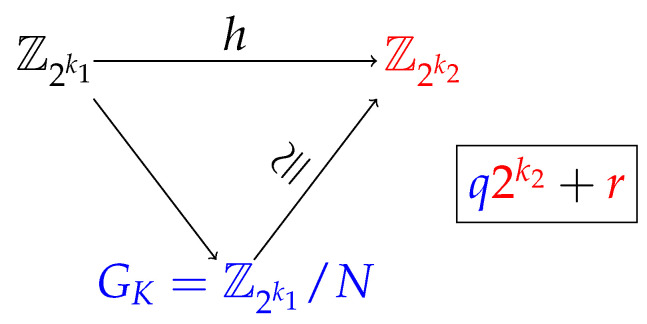
Diagram of the quotient group of the keys.

On the other hand, the TBB methodology is based on the definition and calculation of the keys’ quotient group $G_{{}_{K}}$, whose objective is to partition $Z_{2^{k_{1}}}$ (considering $F_{2}^{k_{1}}≅Z_{2^{k_{1}}}$) into equivalent classes, using the homomorphism *h* defined as follows:

$$\begin{array}{ccc} h:Z_{2^{k_{1}}} & \to & Z_{2^{k_{2}}}\\ a & \mapsto & a\;(\mathrm{mod}\;\;2^{k_{2}}), \end{array}$$

so $G_{{}_{K}}=Z_{2^{k_{1}}}/N$, where *N* is the kernel of *h*. The diagram in Figure 2 presents the structure of $G_{{}_{K}}$ with respect to $Z_{2^{k_{1}}}$ and $Z_{2^{k_{2}}}$.

## 3. About the Closeness Problem

The analysis will focus on the fitness function $F_{4}$, from Equation (3), which measures the fitness of each individual *X* of the key space, comparing the ciphertext *C*, and the text obtained from encrypting *T* with *X*. In short, it measures the decimal distance between ciphertexts. In this sense, the focus is on the problem of verifying if the approximation to 1 of $F_{4}\left(X\right)$ in the comparison of the ciphertexts (that is, the approximation of E(X,T) to C=E(K,T)), implies decimal proximity to the real key *K* being searched for, with which *T* was encrypted to obtain *C*. This problem will be referred to as *Closeness Problem* (CP).

### 3.1. Closeness Strategy

In this section, the first approximation of the CP is proposed. To test it, an attack strategy is proposed that links the two key space partitioning methodologies, BBM and TBB, and will be referred to as the *Closeness Strategy*. We will divide the strategy into three stages, which are detailed below:

1. First, the idea is that, given *T*, *K* and *C*, such that C=E(K,T), choose $k_{2}$ and $k_{d}$ in the TBB methodology and then search for the key *K* in any class of the quotient group of keys $G_{{}_{K}}$ (see [19]). For uniformity, the key will be searched for in the class to which the ciphertext belongs. The purpose at this first moment is not for the GA to find the key directly (that is why the choice of the class could even be random or chosen according to another criterion) but, in the end, to choose the individual of the population with the greatest adaptation, the fittest, returned as a solution by the GA, say $X_{1}$. At this point, the fitness of $X_{1}$, and its decimal distance to *K*, must be calculated: $F_{4}\left(X_{1}\right)$, and, $S_{1}=\left|{X_{1}}_{d}-K_{d}\right|$;
2. Then, partition the space using the BBM methodology (in this case, exchanging the values of $k_{2}$ and $k_{d}$, to perform the search under the same conditions as with the TBB methodology). Select the class in which the fittest individual is found that was obtained as a solution with the TBB methodology in Stage 1 ($X_{1}$). At the end of the GA, the best-fit individual returned is taken as the solution, say, $X_{2}$. As in the previous case, the fitness of $X_{2}$ is taken, and its decimal distance to *K*: $F_{4}\left(X_{2}\right)$, and, $S_{2}=\left|{X_{2}}_{d}-K_{d}\right|$;
3. For the purposes of testing the Closeness Problem, we will say that a better solution was obtained at Stage 2 if the following condition holds,
  (5) $$F_{4}\left(X_{2}\right)>F_{4}\left(X_{1}\right)\;\;\wedge \;\;S_{2}<S_{1}.$$
  That is, if $X_{2}$ is closer to *K* than $X_{1}$, at the same time, it is more suitable.

Note that, when performing the partition with the TBB methodology, each class has individuals from the population distributed throughout the space. In this sense, all the intervals of the BBM methodology have at least one individual of each class taken from TBB. For this reason, the TBB methodology is used first, where the individual with the highest fitness is expected to be closest to the key *K*, according to the decimal distance. Stage 1 is based on this fact.

Then, the idea of Stage 2 is to search for the key in an integer interval around $X_{1}$, with the goal of finding an individual $X_{2}$ that is closest to the key in its decimal place, and at the same time, has a higher fitness value than $X_{1}$. For this purpose, the search is carried out in this stage with the BBM methodology, which partitions into integer intervals (see Section 2.3). The interval to choose is the class to which the individual $X_{1}$ belongs when performing the partition with BBM. Suppose that *q* is the class to which $X_{1}$ belongs in BBM and in which to start searching. So, if one wants to widen the search range, one should take the classes immediately before and after *q*, starting with this one. In other words, it searches successively in the classes,

(6) $$q,\;q\pm 1,\;q\pm 2,\;\ldots ,\;q\pm n,\;n\in Z_{+}^{*},$$

which would be equivalent to progressively increasing the radius of the interval to the desired depth level. As explained above, reversing the order of the methodologies in the Stages 1 and 2 would not make the same sense concerning testing the Closeness Problem and the decimal distance.

Stage 3 is essential for answering the Closeness Problem. Remember that the main objective is to verify if the closeness of the ciphertexts, and, therefore, the tendency to 1 of the fitness function, implies positional decimal closeness of the individual to the real key. Therefore, to say that the result obtained in the second stage is good is not enough to find an individual with greater fitness. Worse still is finding an individual closer to *K*; on the contrary, its adaptation is less than the solution found in the first stage. In the first case in which an individual is found that only complies with having greater fitness, no data are obtained to verify the proximity to *K* since it could be further from it than the individual in the first stage. For this reason, both conditions must be fulfilled simultaneously and, therefore, the relationship in Equation (5).

The importance of the Closeness Problem lies in the fact that we are getting closer to the key, even if it is not known. When performing the attack to search for the key, if it is not found, then the idea is to have a certain degree of certainty that the individual who found the solution is positionally closest to the key.

### 3.2. Applications to Cryptanalysis

For future research, and with processors with higher computing capacity, it would be interesting to test the following attack methodology based on the Closeness Problem and which will be referred to as the *Decimal Closeness Attack* (DCA). The DCA constitutes an application of the results concerning the CP to the attack on block ciphers.

Given *T* and *C* as defined above, the attack’s goal is to find *K* such that E(K,T)=C. The main idea of the DCA is to increase the radius of the search interval around *q* and search for the key with the GAs in those classes. That is, each time Step 1 is applied, Step 2 should be applied several times. The rationale is precise that each time a solution with higher fitness is found, it will also be assumed that it is closer to the key and, therefore, that it satisfies the relationship shown in Equation (5).

Once the experiments were performed, an average reference distance ϵ was calculated, obtained as the average of the distances,

(7) $$S_{2}^{l}=\left|X_{2}^{l}-K^{l}\right|,$$

in the attacks made to each trio $\left(T^{l},K^{l},C^{l}\right),\;l=\bar{1,n},\;n\in Z_{+}$:

(8) $$\epsilon =\left\lceil \frac{\sum_{i=1}^{n}S_{2}^{i}}{n}\right\rceil \cdot$$

In other words, ϵ is the average distance of the solution obtained in the second stage, $X_{2}$, from the key *K*. Assuming this distance in the DCA, the search will also be performed on the two classes, $q_{{}_{1,2}}$, corresponding to the individuals $X_{3,4}=X_{2}\pm \epsilon$:

(9) $$q_{{}_{1,2}}=\frac{\left(X_{2}\pm \epsilon \right)-\left(X_{2}\pm \epsilon \right)\;\left(\mathrm{mod}\;\;2^{k_{2}}\right)}{2^{k_{2}}}.$$

That is, it will not only search for an interval around $X_{2}$, but also around $X_{3}=X_{2}-\epsilon$ and $X_{4}=X_{2}+\epsilon$. The last two cases would be the result of experimentation; the more experiments that are carried out, the more precise the estimate of ϵ will be. In this case, the advantage of the BBM and TBB key space partitioning methodologies is that they allow the search to be performed simultaneously in different classes, saving time in the attack.

To summarize, given the pair (T,C), the DCA consists of the following. Apply Stage 1 and get $X_{1}$. Apply the Stage 2 with the BBM methodology and search the class to which $X_{1}$ belongs to obtain $X_{2}$. Finally, search with the GA around $X_{2}$, $X_{3}$, and, $X_{4}$, that is, in the classes,

(10) $$q\pm i_{0},\;q_{{}_{1}}\pm i_{1},\;q_{{}_{2}}\pm i_{2},\;i_{j}=\bar{0,n_{j}},\;j\in \left\{0,1,2\right\},\;n_{j}\in Z_{+}^{*}.$$

Only five classes were searched, and ϵ is large. However, as the search radius increases around *q* in experiments, ϵ will become smaller. See Section 5 for the experiments with the closeness strategy.

## 4. On the Fitness Functions and the Change Detection

From now on, M, K, and C will be the space for the plaintexts, keys, and ciphertexts, respectively. The purpose is to characterize fitness functions and determine, in advance, whether one fitness function is better than another. Informally, we will say that the fitness function $f_{1}\left(x\right)$ (x∈K) is better than $f_{2}\left(x\right)$, if $f_{1}$ detects more changes in *x* than $f_{2}$. Each change in *x* is detected in different function values each time. For example, given

(11) $$x_{1}<x_{2}<\cdots <x_{10}\in K,$$

if $f_{2}$ remains constant in $x_{1},\ldots ,x_{5}$,

(12) $$f_{2}\left(x_{1}\right)=\cdots =f_{2}\left(x_{5}\right)=a;$$

so it is not detecting changes from $x_{1}$ to $x_{5}$. Therefore, it does not reflect the approach of $x_{1}$ to $x_{5}$. In the extreme case, neither is the closeness to $x_{10}$, despite the fact that $x_{5}$ is closer to $x_{10}$ than $x_{1}$. However, if $f_{1}$ were different in all cases, then it would detect the changes and the closeness of $x_{1}$ to $x_{10}$. This fact causes better behavior of $f_{1}$ concerning $f_{2}$. It is clear that the probabilistic and pseudo-random complexity that both encryption algorithms and GAs have are being overlooked in the above (and later). The focus is only on the structure of the fitness functions since the characteristics of the cryptosystems and the GAs do not depend on them.

The functions $F_{1}$ and $F_{4}$ (see Section 2.2) use two different distances, Hamming’s distance and the decimal distance. There are changes that $F_{1}$ does not detect, unlike $F_{4}$. For example, suppose the key is $a={\left(1,1,1,1,1,1\right)}_{2}$, and $b={\left(0,0,0,0,0,1\right)}_{2}$ is the possible key, both in binary. It is clear that Hamming’s distance is 5, and the decimal distance is 62 since a=63, and b=1; and the fitness functions take the values 1−5/6=0.17 for $F_{1}$ and 1−62/63=0.016 for $F_{4}$. Now, if $b={\left(0,0,1,0,0,0\right)}_{2}$, the function $F_{1}$ would still be 0.17 since there are still five different bits; on the other hand, b=8, so $F_{4}$ takes the value 1−55/63=0.13. Finally, if we take $b={\left(1,0,0,0,0,0\right)}_{2}=32$, then Hamming’s distance remains constant but the decimal keeps changing, so the fitness function does too and takes the value 0.49. Therefore, this shows that the change of *b* is detected by the decimal distance most of the time, contrary to the binary distance, which stays the same over many more changes.

Considering the above, the objective of what is proposed in this section is to start the basis of a theory that allows an explanation of the aforementioned. Let *f* be a fitness function that depends on a distance function *d*; the analysis will focus separately on the characteristics of *f* and *d*, understanding that the results on the distance influence *f* also.

**Definition** **1.** *Given $\delta \in \mathrm{Img}\left(f\right)\subset \left[0,1\right]\subset R_{+}$, we will call the* Completeness Kernel *of f in δ, Com(f,δ), to the set:*

(13) Com(f,δ)={x∈K|f(x)=δ}.

The completeness kernel is a way to obtain a range of elements in which *f* is remained constant and therefore does not reflect changes occurring in the keys. In the example with $f_{2}$,

(14) $$Com\left(f_{2},a\right)=\left\{x_{1},\;x_{2},\;x_{3},\;x_{4},\;x_{5},\ldots \right\}$$

That is, at least it is known that the elements $x_{1},\ldots ,x_{5}$ are in the completeness kernel $Com(f_{2},a)$.

**Definition** **2.** *The* Center of Completeness *of f, Cen(f), is the set,*

(15) Cen(f)={#Com(f,δ)|∀δ∈Img(f)}.

*The Degree of Completeness, $\lambda_{f}$, of f, is the maximum of its center of completeness, $\lambda_{f}=\max\left(Cen\left(f\right)\right)$. Then, f is said to be $\lambda_{f}$-complete.*

The degree of completeness globally measures the worst result of *f* in terms of the number of elements in its completeness kernels. The larger $\lambda_{f}$ is, the less effective *f* is, in the sense that the larger the range in which it detects no change. What is desired is to have fitness functions that are 1-complete.

**Lemma** **1.** *If there is a kernel of completeness of f with cardinality θ, then the degree of completeness of f is greater than or equal to θ. More formally,*

(16) $$f,\;\delta \in \mathrm{Img}\left(f\right),\;\theta \in Z_{+}^{*},\;\exists \;Com\left(f,\delta \right),\;\#Com\left(f,\delta \right)=\theta \Rightarrow \lambda_{f}\geq \theta .$$

**Proof.**  Given a fitness function *f*, suppose there exists Com(f,δ) with cardinality θ, for some value δ∈Img(f). It is clear that θ∈Cen(f), and there are only two possibilities—that it is less than or equal to the maximum of Cen(f), which is equivalent to $\lambda_{f}$—therefore, it must be $\lambda_{f}\geq \theta$. □

It is a hard problem to determine the degree of completeness of a fitness function. This is due, first of all, to the size of the key space. Another point is the very structural complexity of the *E* cipher, which depends on the key, and at the same time, most fitness functions also use *E* in their construction.

The cipher *E* often takes the same value for different keys *x* because the combination of keys and plaintexts is much larger than the cardinality of the ciphertext space. Then, by Dirichlet’s Principle, at least one pair of keys $x_{1},\;x_{2}$, returns the same ciphertext:

(17) $$\exists \;x_{1},x_{2}\in K,T_{1},T_{2}\in M\;\;\left(E\left(x_{1},T_{1}\right)=E\left(x_{2},T_{2}\right)\in C\right).$$

In this sense, it is complicated to ensure higher bounds for $\lambda_{f}$ (other than |K|). This fact influences some fitness functions not detecting the change between $x_{1}$ and $x_{2}$. However, that would not depend on them but on the cipher *E*. In practice, it is a hard problem to determine the pairs $\left(x_{i},T_{i}\right)$ in which equal ciphertext is obtained. The same would happen in the opposite case, where the fitness functions compare the plaintexts from the cryptosystem’s decryption algorithm.

**Definition** **3.** *Let d be a distance function, and, $s\in \left[0,d_{max}\right]\subset Z_{+}$ be the distance between two arbitrary elements of C. We will call the* Plateau *of d at $C_{0}\in C$ with respect to s, the set $M(d,C_{0},s)$ (or simply M(d)):*

(18) $$M\left(d,C_{0},s\right)=\left\{C\in C|\;\exists \;x\in K,T\in M,C=E\left(x,T\right),\;d\left(C,C_{0}\right)=s\right\}.$$

*We will say that $C_{0}$ is the* Axis of the Plateau.

**Definition** **4** (Reduced Plateau). *Let $C_{0}\in C$, d be a distance function, $s\in \left[0,d_{max}\right]\subset Z_{+}$ be the distance between two arbitrary elements of C, and, $M(d,C_{0},s)$ a plateau of d. Two arbitrary elements $C_{i}$, $C_{j}$ of $M(d,C_{0},s)$ are equivalent in $M(d,C_{0},s)$, if they can be obtained with the same keys, i.e.,*

(19) $$C_{i}=E\left(K_{i},T_{i}\right),\;C_{j}=E\left(K_{j},T_{j}\right)\;\;\left(K_{i}=K_{j}\Rightarrow C_{i}\equiv C_{j}\right).$$

*The reduced plateau is the one obtained by eliminating equivalent elements in $M(d,C_{0},s)$, leaving only one representative in each case for each key.*

**Definition** **5** (Maximum plateau). *Let d be a distance function. The maximum plateau of d, $M_{max}\left(d\right)$, is the largest cardinal reduced plateau for all possible axes and values of $s\in \left[0,d_{max}\right]\subset Z_{+}$.*

**Figure 3:**
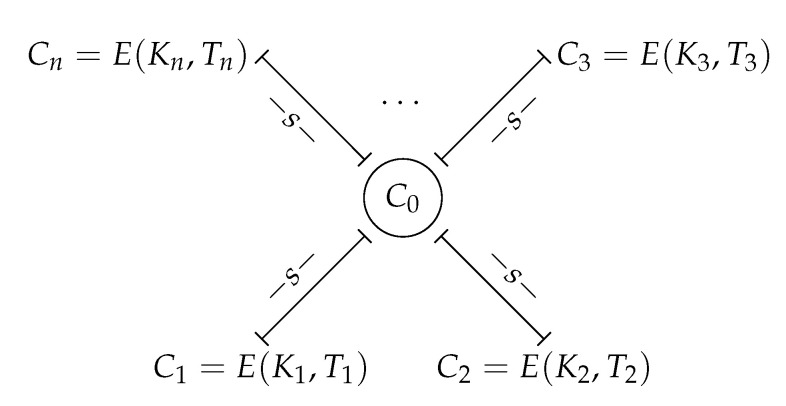
Example of a plateau of cardinality *n*.

Figure 3 shows a schematic example of a plateau of cardinality *n*. In general, the $T_{i},\;i=\bar{1,n}$ can be the same all at once. However, if the plateau were reduced, the keys $K_{i}\in K,\;i=\bar{1,n}$, must be different two by two. The reason is that the analysis of the fitness functions focuses on the changes of the individuals in the GA population, which coincide with the elements of the key space.

The interesting property of the maximum plateau is its cardinal. In this sense, there is no difficulty if several maximum plateaus have the same number of elements.

**Definition** **6.** *Let d be a distance function, and $M^{\left(1\right)}\left(d\right)$ and $M^{\left(2\right)}\left(d\right)$ two reduced plateaus of d. We will say that $M^{\left(1\right)}\left(d\right)$ and $M^{\left(2\right)}\left(d\right)$ are equivalent if they have the same cardinality:*

(20) $$|M^{\left(1\right)}\left(d\right)|=|M^{\left(2\right)}\left(d\right)|\Leftrightarrow M^{\left(1\right)}\left(d\right)\equiv M^{\left(2\right)}\left(d\right).$$

It is clear that if $M^{\left(1\right)}\left(d\right)$ is a maximum plateau, then so is $M^{\left(2\right)}\left(d\right)$.

**Definition** **7** (Degree of detection). *The* Degree of Detection *of a fitness function f is the pair $\left(\lambda_{f},|M_{max}\left(d\right)|\right)$, and will be written simply, $D_{f}^{\left(\lambda_{f},|M_{max}\left(d\right)|\right)}$. The function f is of perfect degree if it is 1-complete and $|M_{max}\left(d\right)|=1$.*

The ideal would be to look for fitness functions for GAs applications whose degree of detection is getting closer and closer to the perfect degree.

**Proposition** **1.** *Given $\alpha_{1},\;\alpha_{2}\in R$, d(x) a distance and f(x) a fitness function with x∈K. If f is of the form*

(21) $$f\left(x\right)=\alpha_{1}+\alpha_{2}d\left(x\right),$$

*and d has a reduced plateau of cardinal ρ, then, $\lambda_{f}\geq \rho$.*

This statement says nothing about the internal structure of *d*.

**Proof.**  Let $\alpha_{1},\;\alpha_{2}\in R$, d(x) be a distance and f(x) be a fitness function with x∈K. Suppose *f* has the form,

(22) $$f\left(x\right)=\alpha_{1}+\alpha_{2}d\left(x\right),$$

and that $M_{max}\left(d,C_{0},s\right)$ is a reduced plateau of *d*, such that, $|M_{max}\left(d,C_{0},s\right)|=\rho$, for some $C_{0}\in C$ and $s\in R_{+}$. By the Definitions 3 and 4, there exist ρ keys $x_{i}\in K$, $i=\bar{1,\rho }$, such that, $d(x_{i})=s$. From the form of *f* in (22), it is clear that *f* is also remained constant and equal to

(23) $$\alpha_{1}+\alpha_{2}s$$

for each of these keys. Therefore, the set,

(24) $$V={\left\{x_{i}\right\}}_{i=1}^{\rho },$$

is a completeness kernel of *f* of cardinal ρ. Then, applying the Lemma 1 with θ=ρ, we obtain, $\lambda_{f}\geq \rho =\left|V\right|$. □

## 5. Experiments and Results

### 5.1. Closeness Strategy

Experiments were carried out with a Laptop Personal Computer with a processor: Intel(R) Celeron(R) CPU N3050 @1.60 GHz (2 CPUs), ∼1.6 GHz, and 4 GB of RAM. The experiment consisted of applying the Closeness Strategy with the function $F_{4}$ to the AES(*t*) encryption for t=3 (AES(*t*) is a parametric version of AES (*Advanced Encryption Standard*), where t∈{3,4,5,6,7,8}, and AES(8) = AES, see [21,22]).

In the case of the AES(3), $k_{1}=48$, $k_{2}=38$, and $k_{d}=10$ were taken in the TBB methodology, and conversely for BBM ($k_{2}=10$ and $k_{d}=38$). With these data, the GA carried out 10 generations. One hundred pairs of plaintexts and keys were randomly generated, and the corresponding 100 ciphertexts were calculated. The strategy was applied to each trio (T,K,C=E(K,T)). In the second stage with the BBM methodology, five classes were searched for: the class *q* of the element $X_{1}$ of the first stage, and the classes

(25) q−1,q+1,q−2,q+2,

which represent an insignificant amount concerning the total number of classes:

(26) $$2^{k_{d}}=2^{38}=274\;\;877\;\;906\;\;944.$$

Although the search interval was small, as a result, a better solution was not obtained in only 12 occasions. Therefore, in 88% of the attempts, the CP was positively verified, finding individuals with greater fitness and, at the same time, closer to the key *K*.

Under the same conditions, the same procedure was applied with the function $F_{1}$. Note that in this case, $F_{1}$ used Hamming’s distance with the binary blocks, and therefore it was totally different from $F_{4}$. If the results behave similarly to $F_{4}$, then it would make no difference whether the distance used was decimal. However, out of 30 attempts, 13 failures had already been obtained, and only 17 positive solutions were found (for a $56.6\bar{6}$% effectiveness). That is, in 30% of attempts with $F_{4}$, the function $F_{1}$ reached $108.3\bar{3}$% of failures. This shows that it is more effective to achieve decimal closeness to the key by using fitness functions that use decimal distance.

### 5.2. Comparison of Two Fitness Functions

We will focus the analysis on the distances of $F_{1}$ and $F_{4}$ to compare these fitness functions using the results from Section 4. These functions can be written in the form (21),

(27) $$\begin{array}{ccc} F_{1}\left(X\right) & = & 1-\frac{1}{n}d_{H}\left(C,E\left(X,T\right)\right), \end{array}$$

(28) $$\begin{array}{ccc} F_{4}\left(X\right) & = & 1-\frac{1}{2^{n}-1}d\left(X\right),\;d\left(X\right)=\left|C_{d},E{\left(X,T\right)}_{d}\right|, \end{array}$$

In the case of $F_{1}$, $d_{H}$ is the Hamming’s distance between binary blocks of length *n*. Take, for reference, the binary null vector of length *n*:

(29) $$O=[0,\;0,\;\underset{n-3}{\underset{︸}{\cdots }},\;0].$$

The vector $C_{1}$,

(30) $$C_{1}=\left[0,\;0,\;\underset{n-4}{\underset{︸}{\cdots }},\;0,\;1\right],$$

has a Hamming’s distance equal to 1 with respect to *O*, $d_{H}\left(O,C_{1}\right)=1$. Now, by varying the 1 in $C_{1}$, a total of *n* different vectors are obtained that maintain a Hamming’s distance equal to 1 with respect to *O*, in which $d_{H}$ does not detect the change. If we take $C_{2}$ with two 1 s:

(31) $$C_{2}=\left[0,\;0,\;\underset{n-5}{\underset{︸}{\cdots }},\;0,\;1,\;1\right],$$

then the Hamming’s distance is, $d_{H}\left(O,C_{2}\right)=2$. In this case, there would be

(32) n2=n!2!(n−2)!=n(n−1)2

different ways to place the two 1s in $C_{2}$ to obtain vectors with equal distance from *O*. Therefore, there are $\frac{n(n-1)}{2}$ different vectors with Hamming’s distance equal to 2. In general, if a vector with *t* 1s was chosen, then there would be nt different vectors with equal distance from *O*:

(33) nt=n!t!(n−t)!,

which would be equivalent to having plateaus whose cardinality would be, at least, equal to that number of vectors. Therefore, to compare $F_{1}$ and $F_{4}$, it is enough to take the degree of completeness, for example, greater than *n*, $\lambda_{F_{1}}\geq n$ (note that there are larger plateaus, as in the case of $C_{2}$, with, $\frac{n(n-1)}{2}\geq n,\;n\geq 3$). Similar reasoning would be obtained if, on the contrary, the vector whose components are all equal to 1 had been taken as a reference.

For $F_{4}$, the distance *d* is the decimal between positive integer values. In this case, taking C∈C with $C_{d}\notin \left\{0,\;2^{n}-1\right\}$, it is clear that, for a given value of the distance *s*, there are only, at most, two values that are at that distance, $C_{d}-s$ and $C_{d}+s$. In other words, it is fulfilled that

(34) $$d\left(C_{d},C_{d}-s\right)=d\left(C_{d},C_{d}+s\right)=s.$$

So the degree of completeness is $\lambda_{F_{4}}\geq 2$. Therefore, there is a greater chance that $F_{4}$ will outperform $F_{1}$. In this sense, in [14], it was already verified that, globally, fitness functions that use decimal distance behave better than those that use Hamming’s distance when the objective is to find the key, making a balance between the time consumed, the number of generations needed on average to find the solution, and the number of times the key was found.

**Figure 4:**
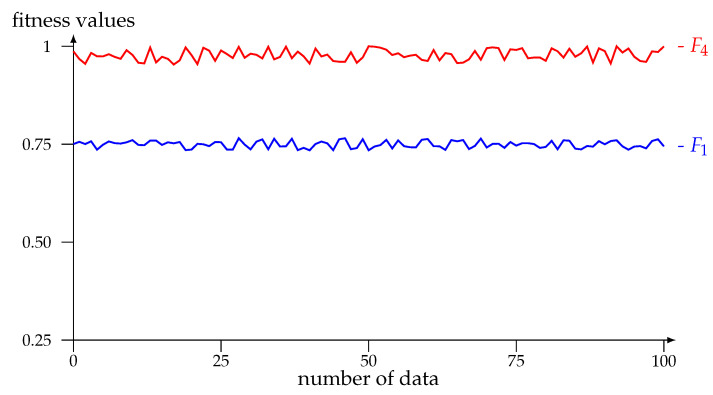
Values of the fitness functions $F_{1}$ and $F_{4}$.

On the other hand, experiments were performed to compare the fitness of the fittest individuals returned as a solution by GA using these fitness functions in cases where the cues were not found. In particular, 100 data points were taken for each of the fitness functions in the same experiments of Section 5.1, whose behavior can be observed in Figure 4.

In these experiments, on average, the fitness of the fittest individuals with $F_{1}$ was approximately ±0.75. With $F_{4}$, the values are greater than or equal to ±0.98 in general, reflecting the better behavior of $F_{4}$. Note that, if the key is found, then the fitness of that individual is 1.

## 6. Conclusions

In the present work, a study was carried out on the fitness functions that intervene in GAs and the attack on block ciphers. First, a methodology called Closeness Strategy was proposed, verifying that the closeness to 1 of the value of some fitness functions that use decimal distance implies decimal closeness to the key. In this direction, the Decimal Closeness Attack was also proposed, the foundation of which is the Closeness Strategy. On the other hand, the basis of a theory that allows the future characterization of the fitness functions and the determination, in advance, if one is more effective than another in the attack on block ciphers using the Genetic Algorithm, is initiated. In this last case, the best behavior of the fitness functions that use decimal distance is corroborated when the objective of the attack is to find the key.

For future work, it is interesting to apply the DCA to attack some ciphers and continue advancing in the characterization of fitness functions according to their degree of detection, as well as developing procedures that allow calculating with greater precision the degree of detection of a fitness function.

## Abbreviations

The following abbreviations are used in this manuscript:

$F_{2}$	The Galois field with two elements
Z	The set of integer numbers
R	The set of real numbers
M	The space of the plaintexts
K	The space of the keys
C	The space of the ciphertexts
*T*, *K*, *C*	A plaintext, key and ciphertext respectively
*d*, $d_{H}$	Decimal and Hamming’s distances respectively
Com(f,δ)	Completeness Kernel of *f* in δ
Cen(f)	Center of Completeness of *f*
$\lambda_{f}$	Degree of Completeness of *f*
$M(d,C_{0},s)$	Plateau of *d* at $C_{0}\in C$ with respect to *s*
$M_{max}\left(d\right)$	Maximum plateau of *d*
$D_{f}^{\left(\lambda_{f},|M_{max}\left(d\right)|\right)}$	Degree of Detection of *f*
$G_{{}_{K}}$	Quotient group of the keys
GA	**G**enetic **A**lgorithm
TSP	**T**raveling **S**alesman **P**roblem
AES	**A**dvanced **E**ncryption **S**tandard
CP	**C**loseness **P**roblem
BBM	Miguel A. **B**orges-Trenard, Mijail **B**orges-Quintana and Lázaro **M**onier-Columbié
TBB	Osmani **T**ito-Corrioso, Miguel A. **B**orges-Trenard and Mijail **B**orges-Quintana
RSA	**R**ivest, **S**hamir and **A**dleman
DES	**D**ata **E**ncryption **S**tandard
DCA	**D**ecimal **C**loseness **A**ttack
